# The Need for Special Education Among ELBW and SGA Preterm Children: A Cohort Study

**DOI:** 10.3389/fped.2021.719048

**Published:** 2021-10-20

**Authors:** Pauline E. van Beek, Kaylee van de Par, Iris E. van der Horst, Anneloes L. van Baar, Brigitte Vugs, Peter Andriessen

**Affiliations:** ^1^Department of Neonatology, Máxima Medical Center, Veldhoven, Netherlands; ^2^Department of Child and Adolescent Studies, Utrecht University, Utrecht, Netherlands; ^3^Department of Psychology, Máxima Medical Center, Veldhoven, Netherlands; ^4^Department of Applied Physics, School of Medical Physics and Engineering, Eindhoven University of Technology, Eindhoven, Netherlands

**Keywords:** neurodevelopmental outcome, very preterm children, special education, very low birth weight, small for gestational age

## Abstract

**Background:** Preterm infants with pre- or postnatal growth restriction may have an additional risk of adverse neurodevelopmental outcome. Whereas reduced cognitive ability and behavioral problems have consistently been associated with prematurity, a more comprehensive evaluation is necessary to identify those preterm infants who are at increased risk for difficulties in school performance. This study evaluated the association between extremely low birth weight (ELBW) and the need for special education and determined if there is an additional risk for the need for special education among small for gestational age (SGA) children.

**Methods:** This is a single-center cohort study including singleton children born below 30 weeks' gestation between 1990 and 2011 and followed into 2019. ELBW + was defined as a birth weight below 1,000 g, which was compared to ELBW–. Within all ELBW+ children, SGA+ was defined as a birth weight <10th percentile according to Fenton, which was compared to SGA–. The dichotomous outcome measurement was the need for special education at 8 years of age or not, reflecting if the children required a special educational setting designed to accommodate educational, behavioral, and/or medical needs.

**Results:** In total, 609 children were eligible for follow-up, of whom 390 (64%) children were assessed at 8 years. Of these, 56 (14%) children needed special education, most often determined by cognitive deficiency (43%), behavioral problems (29%), or both (16%). Among the 191 ELBW+ children, 35 (18%) attended special education, compared to 21 (11%) among ELBW– children (*p*-value 0.041). A decreasing risk for the need for special education was found from 25% in ELBW+/SGA+ children to 16% in ELBW+/SGA– children and 11% in ELBW–/SGA– children (*p*-value 0.025). Multivariable logistic regression showed an odds ratio of 2.88 (95% CI 1.20–6.78) for ELBW+/SGA+ children vs. ELBW–/SGA– children for the need for special education.

**Conclusions:** This study showed that ELBW children are at increased risk for the need for special education compared to non-ELBW children. In addition, children that are both ELBW and SGA do have the highest risk for the need for special education. Classifying children as ELBW and SGA can be useful in follow-up for identifying preterm children with an additional risk for adverse long-term outcome.

## Introduction

Over the last few decades, improvements in perinatal management of very preterm newborns made it necessary to consider the long-term outcome of these infants (1). Very preterm-born children have shown a higher risk for neurosensory disabilities as well as cognitive, motor, behavioral, and academic problems later in life (2–7). Underlying these neurodevelopmental deficits, suboptimal fetal growth is likely to be a key factor (8). A recently published meta-analysis suggested that being small for gestational age (SGA, defined as a birth weight <10th percentile) is associated with an additional risk of adverse neurodevelopmental outcome to that associated with very preterm birth alone (9). The combination of SGA and preterm delivery might additively result in higher rates of perinatal complications and consequently worse long-term neurocognitive outcomes, compared to SGA children delivered at term (10). It has also been shown that, independent of SGA, extremely low birthweight (ELBW, defined as birth weight below 1,000 g) infants are at higher risk for adverse long-term outcome compared to non-low birth weight infants (11).

Whereas, cognitive ability and behavioral problems have consistently been associated with low birth weight, a more comprehensive evaluation is necessary to identify preterm children who are at risk for difficulties in school performance (12). Disorders such as attention deficit hyperactivity disorder, speech-language disorders, and developmental delay are more common among ELBW and SGA children compared to normal birth weight children and do impact school performance (9, 13–16). Due to late recognition of difficulties such as poor concentration and short attention span, sometimes in combination with clumsiness, children might fail in normal schools even in the presence of normal intellectual potential (13, 17).

Therefore, this study aimed to evaluate the association between ELBW and the need for special education and to determine if there is an additional risk for the need for special education among ELBW children that were SGA.

## Methods

### Patient Population

This cohort study included all singleton children born between 1990 and 2011 with a gestational age below 30 completed weeks who were admitted to the neonatal intensive care unit (NICU) of Máxima Medical Centre (MMC), Veldhoven, Netherlands. The NICU of MMC serves a 1.6-million population including antenatal and postnatal transfer from six other hospitals in the region. Children from parents living outside the adherence area of MMC, referrals from other NICUs, and children with congenital malformations were excluded. The ethical review board gave approval for the study and waived informed parental consent for participation in this study.

### Data Collection

In MMC, all preterm children below 30 weeks' gestation were eligible for a follow-up program at the outpatient clinic up to the age of 8 years, including visits to the neonatologist, physiotherapist, and psychologist. Data from the outpatient clinical visits were collected prospectively. Neonatal data were retrieved from the individual medical records. Individual characteristics and medical data included gender (male or female); birth weight in grams; gestational age in days (based on ultrasound findings or on the first day of last menstrual period if ultrasound data was not available); small for gestational age [defined as birth weight below the 10th percentile (18)]; multiplicity (dichotomized as single or multiple birth); mode of delivery (dichotomized as vaginal or by cesarean section); complete course of antenatal corticosteroids (defined as two doses of betamethasone given 24 h apart before the start of labor); Apgar score at 5 min postpartum; inborn or outborn NICU; rate of artificial ventilation >12 h; days of endotracheal intubation on any mode of ventilation; surgical treatment of a persistent ductus arteriosus; intraventricular hemorrhage grade 3 or 4 based on ultrasound (19); cystic periventricular leukomalacia grade 3 (20); severe brain injury (defined as intraventricular hemorrhage grade 3 or 4 or cystic periventricular leukomalacia grade 3); laparotomy for necrotizing enterocolitis or single intestinal perforation; surgical treatment or laser therapy for retinopathy of prematurity; and total days of NICU admission. Socio-economic status was assessed using scores defined by the Netherlands Institute for Social and Cultural Research (The Hague, Netherlands*)* based on postal code at birth, with an average score of 0 and a positive score reflecting a higher-than-average status and a negative score reflecting a lower-than-average status (21). Information on educational status of the parents was collected at follow-up visits. This was classified according to the International Standard Classification of Education 2011 (22). The information was dichotomized, describing whether there was a low education (less than post-secondary education) or at least for one of the parents middle-to-high education (post-secondary education or higher).

### Definition of ELBW and SGA

ELBW was defined as birth weight below 1,000 g, which was compared to non-ELBW defined as a birth weight of 1,000 g or higher. Within all ELBW children, SGA was defined as a birth weight <10th percentile for corresponding gestational age and gender according to Fenton (23), which was compared to non-SGA defined as a 10th birth weight percentile or higher.

### Outcome Measurement

The primary, dichotomous outcome measurement was of the need for special education at 8 years of age or not, reflecting if the children required an educational setting designed to accommodate educational, behavioral, and/or medical needs that could not be adequately addressed in a regular school environment. For each child attending special education, the reason why a child was placed in special school was determined based on the main issue causing problems in regular school based on the anamnesis with parents.

### Statistical Analysis

For this study, ELBW children were compared with non-ELBW children. Within the ELBW children, SGA children were compared with non-SGA children. ELBW vs. non-ELBW and SGA vs. non-SGA children were compared using the Student's *T*-test or Mann–Whitney *U*-test for continuous variables and using the chi-square test for categorical and dichotomous variables. Special education rates were compared between the three groups of ELBW–, ELBW+/SGA–, and ELBW+/SGA+ children using a chi-square test. If significant, additional pair-wise chi-square tests were performed. A logistic regression analysis was performed for the need for special education, including gender, gestational age, parental education, and severe brain injury as parameters in the multivariable model. No data was missing, except for parental education for 25% of the children, which was imputed using the R multivariate imputation by chained equation (MICE) package. A *p*-value < 0.05 was considered significant. Calculations were performed using R version 3.5.1.

## Results

### Study Population and Follow-Up Rates

**Figure 1:**
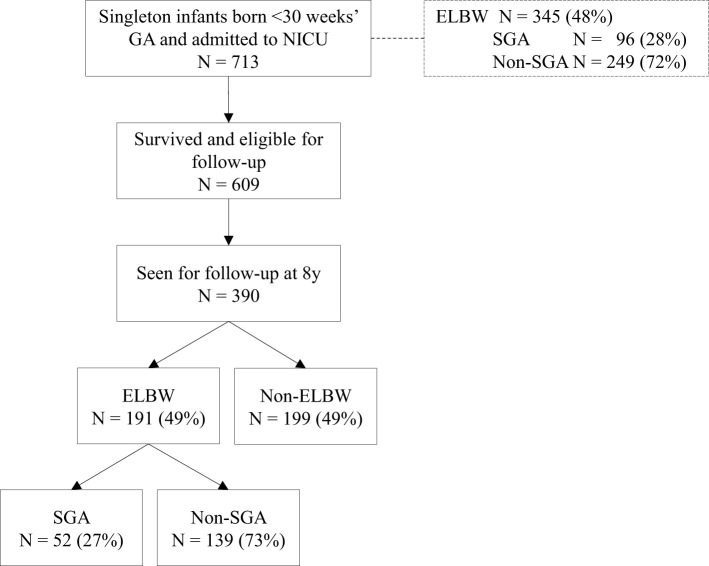
Flowchart of the included children.

Within the study period, 713 singleton children born below 30 weeks' gestational age (GA) were admitted to the NICU, of whom 345 (48%) children were ELBW, of whom 96 (28%) were SGA (Figure 1). Of the 713 admitted children, 609 (85%) children survived and were eligible for follow-up at the outpatient clinic, of whom 390 (64%) children were seen for follow-up at 8 years. Children not seen for follow-up were more mature compared to the children who participated, and their NICU admission lasted significantly shorter (Supplementary Material). Among the children seen for follow-up, distribution of ELBW and SGA was similar to children admitted (ELBW 49%, SGA 27%).

### Baseline Characteristics

Table 1 shows the baseline characteristics for all 390 children seen for follow-up at 8 years, comparing 191 ELBW+ with 199 ELBW– children and 52 SGA+ with 139 SGA– children. ELBW+ and SGA+ children were born with lower birth weights and were more often born by cesarean section, compared to ELBW– and SGA– children. For ELBW+ children, their NICU admissions lasted significantly longer with more complications compared to ELBW– children.

**Table 1:** Baseline characteristics.

				**ELBW+**			**Overall *p*-value**
	**ELBW+**	**ELBW–**	***p*** **-value**	**SGA+**	**SGA–**	***p*** **-value**	
	191 (49%)	199 (51%)		52 (27%)	139 (73%)		
Gender (% male)	88 (46)	120 (60)	0.007^*^	29 (56)	59 (42)	0.139	0.005^*^
Birth weight	823 (125)	1,243 (183)	<0.001^*^	701 (102)	867 (101)	<0.001^*^	<0.001^*^
Gestational age (days)	27.6 [26.5, 28.8]	28.7 [27.0, 29.3]	<0.001^*^	28.4 [27.9, 29.3]	27.3 [26.3, 28.4]	<0.001^*^	<0.001^*^
Gestational age <28 weeks	106 (56)	53 (27)	<0.001^*^	15 (29)	91 (66)	<0.001^*^	<0.001^*^
Caesarean section [*N* (%)]	130 (68)	77 (39)	<0.001^*^	51 (98)	79 (57)	<0.001^*^	<0.001^*^
Antenatal corticosteroids completed [*N* (%)]	131 (69)	107 (54)	0.004^*^	31 (60)	100 (72)	0.145	0.003^*^
Apgar 5 min	8 [6, 9]	8 [7, 9]	0.007^*^	8 [7, 9]	8 [6, 9]	0.062	0.005^*^
Inborn [N (%)]	178 (93)	178 (89)	0.258	52 (100)	126 (91)	0.050	0.053
Socio-economic status	0.02 (0.78)	0.11 (0.79)	0.283	−0.03 (0.89)	0.04 (0.73)	0.557	0.475
Low parental education [*N* (%)]	35 (18)	25 (13)	0.151	12 (23)	23 (17)	0.408	0.583
Ventilation >12 h [N (%)]	145 (76)	115 (58)	<0.001^*^	36 (69)	109 (78)	0.258	<0.001^*^
Days ventilation	5 [1, 12]	2 [0, 5]	<0.001^*^	4 [0, 10]	5 [2, 12]	0.229	<0.001^*^
Surgically treated PDA [*N* (%)]	16 (8.4)	5 (2.5)	0.019^*^	2 (3.8)	14 (10)	0.276	0.009^*^
Severe brain injury	10 (5.2)	12 (6.0)	0.904	2 (3.8)	8 (5.8)	0.871	0.829
Laparotomy	6 (3.1)	2 (1.0)	0.258	1 (1.9)	5 (3.6)	0.901	0.254
Laser therapy for ROP	7 (3.7)	1 (0.5)	0.065	4 (7.7)	3 (2.2)	0.168	0.005^*^
Days NICU	44 [31, 57]	25 [15, 38]	<0.001^*^	44 [33, 56]	45 [31, 57]	0.749	<0.001^*^

*Legend: N (%), mean (SD), or median [1st quartile, 3rd quartile]. The left part of the table shows baseline characteristics for ELBW+ vs. ELBW– children. The right part of the table shows the baseline characteristic for all ELBW+ children, separately for SGA+ and SGA– children. The rightmost column shows the p-value comparing baseline characteristics between the three groups ELBW–, ELBW+/SGA+, and ELBW+/SGA– children. ELBW, extremely low birth weight; SGA, small for gestational age; PDA, patent ductus arteriosus; ROP, retinopathy of prematurity; NICU, neonatal intensive care unit*.

* *Significant on a p-level of 0.05*.

### The Need for Special Education

In total, 56 (14%) children needed special education at the age of 8 years. The need for special education was most often determined by cognitive deficiency (43%), behavioral problems (29%), or both (16%) (Table 2).

**Table 2:** Reasons for attending special education.

**Primary reason for attending special education**	***N* (%)**
Cognitive deficiency	24 (43)
Behavioral problems	16 (29)
Cognitive deficiency and behavioral problems	9 (16)
Cerebral palsy	5 (8.9)
Severely multiply impaired	1 (1.8)
Unclear	1 (1.8)

*Cognitive deficiency and behavioral problems were defined as present if there was a deviation of more than one standard deviation*.

Of the 56 children with the need for special education at the age of 8 years, 21 were ELBW– and 35 were ELBW+, of whom 22 were SGA+ and 13 were SGA–. This resulted in the need for special education among 18% (35/191) ELBW+ children, which was significantly more than the 11% (21/199) among ELBW– children (*p*-value 0.041). A significant decreasing risk for the need for special education was found from 25% in ELBW+/SGA+ children to 16% in ELBW+/SGA– children and 11% in ELBW– children (*p*-value 0.025). A *post-hoc* analysis showed a significant difference between ELBW+/SGA+ and ELBW– children (*p*-value 0.013).

A logistic regression analysis was performed to evaluate the association of being ELBW and SGA with the need for special education, corrected for gender, GA, parental education, and severe brain injury (Table 3). The associations found with univariable analysis remained significant when correcting for the aforementioned factors, with an odds ratio of 2.88 (95% CI 1.20–6.78) for ELBW+/SGA+ vs. ELBW– children for the need for special education. Moreover, female gender and higher gestational age were associated with lower odds for attending special education, while the presence of severe brain injury and low parental education were significantly associated with higher odds for the need for special education.

**Table 3:** Logistic regression for attendance of special education at 8 years.

	**Odds ratio (95% CI)**
SGA/ELBW (ref = ELBW–/SGA–)
ELBW+/SGA–	1.41 (0.65–3.08)
ELBW+/SGA+	2.88 (1.20–6.78)^*^
Gender (ref = male)	0.23 (0.11–0.45)^*^
GA (days)	0.96 (0.92–0.99)^*^
Low parental education	3.55 (1.72–7.23)^*^
Severe brain injury	6.85 (2.33–19.9)^*^

*ELBW, extremely low birth weight; SGA, small for gestational age; GA, gestational age*.

* *Significant on a p-level of 0.05*.

## Discussion

In this large cohort of very preterm children, the association between ELBW and of the need for special education was evaluated, and it was determined if there is an additional risk for the need for special education among ELBW children that were SGA. This study showed that ELBW+ children are at increased risk for the need for special education compared to ELBW– children and that among ELBW+ children, those that were SGA+ do have the highest risk for the need for special education.

Overall, a special education participation rate of 14% was found among preterm-born children at the age of eight. This is consistent with a former Dutch study, showing a special educational rate of 14% among children born at a gestational age of 26–32 weeks. Also, the EPICure study shows a special education rate of 13% among extremely preterm infants (25, 26). These rates are substantially higher than the 1.9–2.7% of children who are enrolled in special primary education between 4 and 12 years throughout the Netherlands in the past 20 years (27).

At 8 years of age, a significant difference was seen in the special education attendance rate between ELBW+ (18%) and ELBW– (11%) children. Within the ELBW+ children, a distinction could be made between SGA+ and SGA– children. A decreasing risk for participation in special education was found from 25% in ELBW+/SGA+ children to 16% in ELBW+/SGA– children toward 11% in ELBW–/SGA– children. The higher rate among SGA+ children compared to SGA– children is in line with the previous Dutch POPS cohort, a nationwide study cohort of very preterm children born alive in 1983 in the Netherlands, which showed that at 9 years of age more SGA children (16.4%) needed special education compared to AGA children (11.9%) (28). The French EPIPAGE study showed school difficulties at 8 years in 28% of the very preterm children born SGA vs. 18% in very preterm children born AGA, which are similar rates to our study (1). It suggests that the effects of SGA remains important even at very low GAs. In addition to the increased risk of special education among ELBW children, SGA children are at the highest risk for special educational needs.

Logistic regression showed that, after correcting for gender, GA, parental education, and severe brain injury, there was still a significant increase for the need for special education for ELBW+/SGA+ children compared to ELBW–/SGA– children. We found that ELBW SGA children had a more than two times higher odds on in the need for special education compared to ELBW–/SGA– children. Moreover, gender, severe brain injury, and low parental education were important factors associated with the need for special educational. Male gender has often been found to be associated with adverse impaired long-term outcome after preterm birth (24, 29–31). Socioeconomic disadvantage does not only increase the likelihood of adverse school performance but is also a risk factor for low birth weight and preterm birth, placing the infant at dual risk from both biological and environmental factors (25).

It was found that most children participated in special education due to cognitive and/or behavioral problems. Although an underlying general cognitive deficit accounted for much of the educational underachievement observed, IQ scores did not account for all of the learning difficulties found in these children (26). Academic performance and behavioral problems such as attention deficit disorders are therefore useful in developmental follow-up in addition to gross IQ measures (25). Extensive neuropsychological testing might be considered in the high-risk group of ELBW+/SGA+ children. In this study, none of the children participated in special education because of blindness or deafness, as none of the children seen for follow-up at 8 years in our cohort were deaf or blind. Although deafness and blindness seldom occur among preterm children, children with such severe hearing or vision problems often drop-out from follow-up as they are already followed in rehabilitation clinics.

Several differences in baseline characteristics between ELBW+ and ELBW– children were observed. These differences were mainly associated with the immaturity of the ELBW+ group. Obviously, this resulted in a longer length of stay of the ELBW infants in the unfavorable NICU environment, which might interfere with postnatal growth and development (32, 33).

Academic performance is associated with long-term health and life chances (34). A major question for parents of a preterm child is whether their child will be able to follow a regular educational trajectory. This study provides an insight that both ELBW and SGA are useful indicators for higher risk of attending special education. This can be useful in follow-up for identifying preterm children with an additional risk for adverse long-term outcome.

Strengths of our paper included the size of the cohort and the outcome measurement at later age. However, this study has several limitations. Our follow-up rate was 64%, which is comparable to follow-up rates at 8 years of age presented in other studies (1). Several studies have found that children lost to follow-up are more likely to have a disability. However, our results might present a higher-risk subset of children as medium-risk children have not always been invited for follow-up, resulting in the fact that children not seen for follow-up were more mature at birth compared to children included in this study. The proportion of ELBW and SGA children remained similar in children seen for follow-up, compared to children admitted to the NICU, and socio-economic status was comparable between children seen and not seen for follow-up. Overall, we expect a low risk of bias induced in the associations observed between being ELBW or SGA and the need for special education.

When evaluating long-term adverse outcome in relation to birth weight, the smallest children may be expected to be at greatest risk. This study aimed to evaluate absolute birth weight and birth weight percentile in relation to the need for special education. It showed that ELBW children are at increased risk for the need for special education compared to non-ELBW children and that among ELBW children, those that were SGA do have the highest risk for the need for special education. Classifying children as ELBW or SGA can be useful in follow-up for identifying preterm children with an additional risk for adverse long-term outcome. Extra attention and more extensive follow-up might be required for the very high-risk group of ELBW+/SGA+ children.

## Data Availability Statement

The datasets presented in this article are not readily available because of privacy regulations according to the General Data Protection Regulation (GDPR). Requests to access the datasets should be directed to Pauline E. van Beek, pauline.van.beek@mmc.nl.

## Ethics Statement

The studies involving human participants were reviewed and approved by Medical Ethics Committee Máxima MC. Written informed consent from the participants' legal guardian/next of kin was not required to participate in this study in accordance with the national legislation and the institutional requirements.

## Author Contributions

PB, AB, BV, and PA contributed to the conception of the study. PB, KP, IH, and BV organized the database. PB performed the statistical analysis and wrote the first draft of the manuscript. PA was responsible for the financial funding of the project and overall supervision. All authors contributed to the interpretation of the results, critically reviewed the manuscript, and approved the submitted version.

## Funding

PB was supported by an unrestricted grant from *Stichting Tiny* & *Anny van Doorne Fonds*. The funding source had no role in the design, conduct, analyses, or reporting of the study or in the decision to submit the manuscript for publication.

## Conflict of Interest

The authors declare that the research was conducted in the absence of any commercial or financial relationships that could be construed as a potential conflict of interest.

## Publisher's Note

All claims expressed in this article are solely those of the authors and do not necessarily represent those of their affiliated organizations, or those of the publisher, the editors and the reviewers. Any product that may be evaluated in this article, or claim that may be made by its manufacturer, is not guaranteed or endorsed by the publisher.

## Abbreviations

NICU: neonatal intensive care unit
GA: gestational age
ELBW: extremely low birth weight
SGA: small for gestational age.

## References

[1] Guellec I Lapillonne A Renolleau S Charlaluk ML Roze JC Marret S. Neurologic outcomes at school age in very preterm infants born with severe or mild growth restriction. Pediatrics. (2011) 127:883. 10.1542/peds.2010-2442 21382951

[2] Torchin H Morgan AS Ancel PY. International comparisons of neurodevelopmental outcomes in infants born very preterm. Semin Fetal Neonatal Med. (2020) 25:101109. 10.1016/j.siny.2020.101109 32354556

[3] Marlow N Wolke D Bracewell MA Samara M EPICure Study Group. Neurologic and developmental disability at six years of age after extremely preterm birth. N Engl J Med. (2005) 352:9–19. 10.1056/NEJMoa041367 15635108

[4] Anderson P Doyle LW Victorian Infant Collaborative Study Group. Neurobehavioral outcomes of school-age children born extremely low birth weight or very preterm in the 1990s. JAMA. (2003) 289:3264–72. 10.1001/jama.289.24.3264 12824207

[5] Linsell L Johnson S Wolke D O'Reilly H Morris JK Kurinczuk JJ. Cognitive trajectories from infancy to early adulthood following birth before 26 weeks of gestation: a prospective, population-based cohort study. Arch Dis Child. (2018) 103:363–70. 10.1136/archdischild-2017-313414 29146572 PMC5890637

[6] Linsell L Johnson S Wolke D Morris J Kurinczuk JJ Marlow N. Trajectories of behavior, attention, social and emotional problems from childhood to early adulthood following extremely preterm birth: a prospective cohort study. Eur Child Adolesc Psychiatry. (2019) 28:531–42. 10.1007/s00787-018-1219-8 30191335 PMC6445809

[7] Twilhaar ES de Kieviet JF Aarnoudse-Moens CS van Elburg RM Oosterlaan J. Academic performance of children born preterm: a meta-analysis and meta-regression. Arch Dis Child Fetal Neonatal Ed. (2018) 103:F322–30. 10.1136/archdischild-2017-312916 28847871 PMC6047144

[8] Miller SL Huppi PS Mallard C. The consequences of fetal growth restriction on brain structure and neurodevelopmental outcome. J Physiol. (2016) 594:807–23. 10.1113/JP271402 26607046 PMC4753264

[9] Sacchi C Marino C Nosarti C Vieno A Visentin S Simonelli A. Association of intrauterine growth restriction and small for gestational age status with childhood cognitive outcomes: a systematic review and meta-analysis. JAMA Pediatr. (2020) 44:122–30. 10.1001/jamapediatrics.2020.1097 32453414 PMC7251506

[10] Bassan H Stolar O Geva R Eshel R Fattal-Valevski A Leitner Y. Intrauterine growth-restricted neonates born at term or preterm: how different? Pediatr Neurol. (2011) 44:122–30. 10.1016/j.pediatrneurol.2010.09.012 21215912

[11] Claas MJ Bruinse HW Koopman C van Haastert IC Peelen LM de Vries LS. Two-year neurodevelopmental outcome of preterm born children. Arch Dis Child Fetal Neonatal Ed. (2011) 96:F169–77. 10.1136/adc.2009.174433 20530098

[12] Eves R Mendonca M Bartmann P Wolke D. Small for gestational age-cognitive performance from infancy to adulthood: an observational study. BJOG. (2020) 127:1598–606. 10.1111/1471-0528.16341 32479707

[13] Hille ET den Ouden AL Bauer L van den Oudenrijn C Brand R Verloove-Vanhorick SP. School performance at nine years of age in very premature and very low birth weight infants: perinatal risk factors and predictors at five years of age. Collaborative project on preterm and small for gestational age (POPS) infants in the Netherlands. J Pediatr. (1994) 125:426–34. 10.1016/S0022-3476(05)83290-1 8071753

[14] Leitner Y Fattal-Valevski A Geva R Eshel R Toledano-Alhadef H Rotstein M. Neurodevelopmental outcome of children with intrauterine growth retardation: a longitudinal, 10-year prospective study. J Child Neurol. (2007) 22:580–7. 10.1177/0883073807302605 17690065

[15] Yu B Garcy AM. A longitudinal study of cognitive and educational outcomes of those born small for gestational age. Acta Paediatr. (2018) 107:86–94. 10.1111/apa.13993 28712154 PMC5763381

[16] Levine TA Grunau RE McAuliffe FM Pinnamaneni R Foran A Alderdice FA. Early childhood neurodevelopment after intrauterine growth restriction: a systematic review. Pediatrics. (2015) 135:126–41. 10.1542/peds.2014-1143 25548332

[17] Ross G Lipper EG Auld PA. Social competence and behavior problems in premature children at school age. Pediatrics. (1990) 86:391–7. 10.1016/S0022-3476(05)82829-X 2388788

[18] Hoftiezer L Hof MHP Dijs-Elsinga J Hogeveen M Hukkelhoven CWPM. From population reference to national standard: new and improved birthweight charts. Am J Obstet Gynecol. (2019) 220:383.e1-383.e17. 10.1016/j.ajog.2018.12.023 30576661

[19] Papile LA Burstein J Burstein R Koffler H Koops B. Relationship of intravenous sodium bicarbonate infusions and cerebral intraventricular hemorrhage. J Pediatr. (1978) 93:834–6. 10.1016/S0022-3476(78)81096-8 568656

[20] de Vries LS Eken P Dubowitz LM. The spectrum of leukomalacia using cranial ultrasound. Behav Brain Res. (1992) 49:1–6. 10.1016/S0166-4328(05)80189-5 1388792

[21] SCP Statusscores. (2017). Available online at: http://www.scp.nl/Formulieren/Statusscores_opvragen (accessed March 1, 2019).

[22] Statistics Netherlands. Education Level. Available online at: https://www.cbs.nl/en-gb/news/2018/20/well-being-not-distributed-equally/education-level (accessed December 24, 2020).

[23] Fenton TR Kim JH. A systematic review and meta-analysis to revise the Fenton growth chart for preterm infants. BMC Pediatr. (2013) 13:59. 10.1186/1471-2431-13-59 23601190 PMC3637477

[24] Wood NS Costeloe K Gibson AT Hennessy EM Marlow N Wilkinson AR. The EPICure study: associations and antecedents of neurological and developmental disability at 30 months of age following extremely preterm birth. Arch Dis Child Fetal Neonatal Ed. (2005) 90:134. 10.1136/adc.2004.052407 15724037 PMC1721849

[25] Schaap AH Wolf H Bruinse HW Smolders-de Haas H van Ertbruggen I Treffers PE. School performance and behaviour in extremely preterm growth-retarded infants. Eur J Obstet Gynecol Reprod Biol. (1999) 86:43–9. 10.1016/S0301-2115(99)00041-X 10471141

[26] Johnson S Hennessy E Smith R Trikic R Wolke D Marlow N. Academic attainment and special educational needs in extremely preterm children at 11 years of age: the EPICure study. Arch Dis Child Fetal Neonatal Ed. (2009) 94:283. 10.1136/adc.2008.152793 19282336

[27] Centraal Bureau voor de Statistiek. Deelname Onderwijs. Available online at: https://jmopendata.cbs.nl/#/JM/nl/dataset/71010ned/line?dl=531C5 (accessed March 20, 2021).

[28] Kok JH den Ouden AL Verloove-Vanhorick SP Brand R. Outcome of very preterm small for gestational age infants: the first nine years of life. Br J Obstet Gynaecol. (1998) 105:162–8. 10.1111/j.1471-0528.1998.tb10046.x 9501780

[29] Hintz SR Kendrick DE Vohr BR Kenneth Poole W Higgins RD Nichd Neonatal Research Network. Gender differences in neurodevelopmental outcomes among extremely preterm, extremely-low-birthweight infants. Acta Paediatr. (2006) 95:1239–48. 10.1080/08035250600599727 16982497

[30] Wolke D Samara M Bracewell M Marlow N EPICure Study Group. Specific language difficulties and school achievement in children born at 25 weeks of gestation or less. J Pediatr. (2008) 152:256–62. 10.1016/j.jpeds.2007.06.043 18206699

[31] Morsing E Asard M Ley D Stjernqvist K Marsal K. Cognitive function after intrauterine growth restriction and very preterm birth. Pediatrics. (2011) 127:874. 10.1542/peds.2010-1821 21382944

[32] Silva LV Araujo LB Azevedo VMGO. Assessment of the neuropsychomotor development in the first year of life of premature infants with and without bronchopulmonary dysplasia. Rev Bras Ter Intensiva. (2018) 30:174–80. 10.5935/0103-507X.20180023 29995082 PMC6031416

[33] Raz S DeBastos AK Newman JB Peters BN Heitzer AM Piercy JC. Physical growth in the neonatal intensive-care unit and neuropsychological performance at preschool age in very preterm-born singletons. J Int Neuropsychol Soc. (2015) 21:126–36. 10.1017/S1355617715000077 25740098

[34] Jansen L Peeters-Scholte C Bruine SW van den Berg-Huysmans A van Klink J van Steenis A. Classroom-evaluated school performance at nine years of age after very preterm birth. Early Hum Dev. (2019) 140:104834. 10.1016/j.earlhumdev.2019.104834 31671378

