# Supplementary material for: The Need for Special Education Among ELBW and SGA Preterm Children: A Cohort Study
Source: Front Pediatr. 2021 Oct 20;9:719048. doi: 10.3389/fped.2021.719048 (PMC8564376; doi:10.3389/fped.2021.719048)
Supplement: Supplementary file 1 [file Table_1.docx]

**Supplementary Material**

|  | **Seen for follow-up** | **Not seen for follow-up** | **p-value** |
| --- | --- | --- | --- |
|  | 390 (64%) | 219 (36%) |  |
| Gender (% male) | 208 (53.3) | 129 (58.9) | 0.214 |
| Birth weight | 1037 (263) | 1099 (285) | 0.007* |
| Gestational age (days) | 198 [190, 204] | 200 [194, 206] | 0.006* |
| Gestational age <28 weeks | 159 (40.8) | 64 (29.2) | 0.006* |
| Caesarean section (N (%)) | 207 (53.1) | 102 (46.6) | 0.145 |
| Antenatal corticosteroids completed (N (%)) | 238 (61.0) | 134 (61.2) | 1.000 |
| Apgar 5 min | 8 [7, 9] | 8 [7, 9] | 0.454 |
| Inborn (N (%)) | 356 (91.3) | 200 (91.3) | 1.000 |
| Socio-economic status (N (%)) | 0.07 (0.78) | -0.05 (0.85) | 0.082 |
| Ventilation >12h (N (%)) | 260 (66.7) | 138 (63.0) | 0.412 |
| Days ventilation | 3 [0, 8] | 3 [0, 8] | 0.566 |
| Surgically treated PDA (N (%)) | 21 (5.4) | 11 (5.0) | 1.000 |
| Severe brain injury | 22 (5.6) | 11 (5.1) | 0.922 |
| Laparotomy | 8 (2.1) | 11 (5.0) | 0.073 |
| Laser therapy for ROP | 8 (2.1) | 3 (1.4) | 0.778 |
| Days NICU | 34 [20, 48] | 25 [14, 44] | <0.001* |

Legend: N (%), mean (SD) or median [1^st^ quartile, 3^rd^ quartile]. PDA = patent ductus arteriosus; ROP = retinopathy of prematurity; NICU = neonatal intensive care unit. * = significant on a p-level of 0.05.
